# Colon-targeting mutual prodrugs of 5-aminosalicylic acid and butyrate for the treatment of ulcerative colitis

**DOI:** 10.1039/c7ra13011b

**Published:** 2018-01-11

**Authors:** Yan Yan, Jinyao Sun, Xianting Xie, Pengchong Wang, Ying Sun, Yalin Dong, Jianfeng Xing

**Affiliations:** School of Pharmacy, Xi'an Jiaotong University 76 Yanta West Road Xi'an Shaanxi 710061 China xajdxjf@mail.xjtu.edu.cn +86-29-82655139 +86-29-82655139; Department of Pharmacy, The First Affiliated Hospital of Xi'an Jiaotong University Xi'an Shaanxi China

## Abstract

The aim of this study was to design and synthesize four colon-targeting mutual prodrugs of 5-aminosalicylic acid (5-ASA) and butyrate, and evaluate their therapeutic effects on ulcerative colitis. Herein, 5-ASB, 5-ASDB, Ols-DB and Ols-DBP were prepared and characterized, and their lipophilicity, solubility, *in vitro* and *in vivo* stability were investigated. Finally, the ameliorative effects of the prodrugs on experimental colitis were evaluated *via* a series of indicators, including the body weight and survival rates of mice, the colon index and colonic damage score, the disease activity index, the myeloperoxidase activity and levels of superoxide dismutase, malondialdehyde, glutathione and glutathione peroxidase in colonic tissues. As a result, 5-ASB was very stable but Ols-DB showed extreme instability in the environment of the gastrointestinal tract, while 5-ASDB and Ols-DBP showed desirable colon-targeting properties. The four prodrugs all had certain therapeutic effects on the experimental colitis. When orally administered to mice, 5-ASDB and Ols-DBP had significantly greater effects than the mixture of 5-ASA and sodium butyrate. Ols-DB was used as an enema and could be as effective as 5-ASDB and Ols-DBP. In addition, the therapeutic effects of the synthesized prodrugs might be associated with their anti-oxidative damage ability.

## Abstract 2

The aim of this study was to design and synthesize four colon-targeting mutual prodrugs of 5-aminosalicylic acid (5-ASA) and butyrate, and evaluate their therapeutic effects on ulcerative colitis.

## Graphical abstract

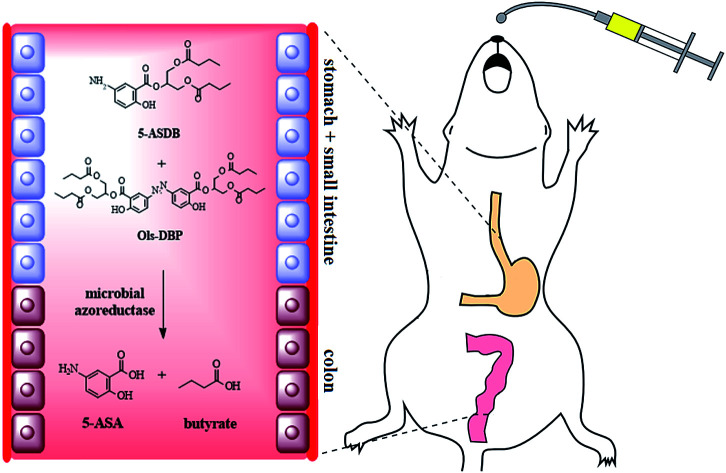

## 1 Introduction

Inflammatory bowel disease (IBD), usually referred to as ulcerative colitis (UC) or Crohn's disease, is a chronic inflammation in the gut.^1^ UC is one of the most common chronic inflammatory forms of IBD and features diffuse mucosal inflammation, mainly limited to the large intestine and colon, and is characterized by diarrhea, rectal bleeding and abdominal pain.^2^ The pathogenesis of UC is obscure and is considered to involve multifactorial interactions amongst genetic, immunological and environmental triggers. During the past decades, therapy for UC has revolved around the non-steroidal anti-inflammatory drug 5-aminosalicylic acid (5-ASA), high and repeated doses of 5-ASA are proven to be necessary for maintenance and preventative therapy of UC relapse.^3^

5-ASA is widely used for mild to moderate active IBD and has become a standard therapeutic drug based on an extensive and long treatment history. However, 5-ASA is rapidly and completely absorbed from the upper intestine when administered orally, but poorly absorbed from the colon.^4^ Free 5-ASA undergoes rapid and nearly complete systemic absorption from the proximal intestine depending on concentration and local pH, which limits its clinical application.^5^ It is thus of tremendous importance to deliver 5-ASA locally to reduce influences by systemic drug absorption causing side-effects and increase the probability for a therapeutic success. Efforts so far have been made for developing oral preparations of 5-ASA. The prodrug approach is an effective way to target the colon as reflected in the clinical success of osalazine.^6^

Short chain fatty acids (SCFAs) are a subset of saturated fatty acids containing six or less carbon molecules, which are the main bacterial metabolites produced following the fermentation of dietary fiber and resistant starches by specific colonic anaerobic bacteria.^7^ SCFAs, especially butyrate, are key promoters of colonic heath and integrity. Butyrate is the major and preferred metabolic substrate for colonocytes providing most of their energy requirements necessary for their proliferation and differentiation.^8,9^ Besides, butyrate performs various physiological functions including anti-inflammatory, antimicrobial, antitumorigenic and immunological roles both locally and systemically in the gut. Vernia *et al.* for the first time reported that oral butyrate is safe and may favor the mucosal repair processes and improve the efficacy of oral 5-ASA in active UC patients.^10^ D'Argenio *et al.* first suggested that given in combination with 5-ASA and sodium butyrate is an effective tool in UC treatment, and the therapeutic effect is significantly greater than both drugs.^11^

**Scheme 1:**
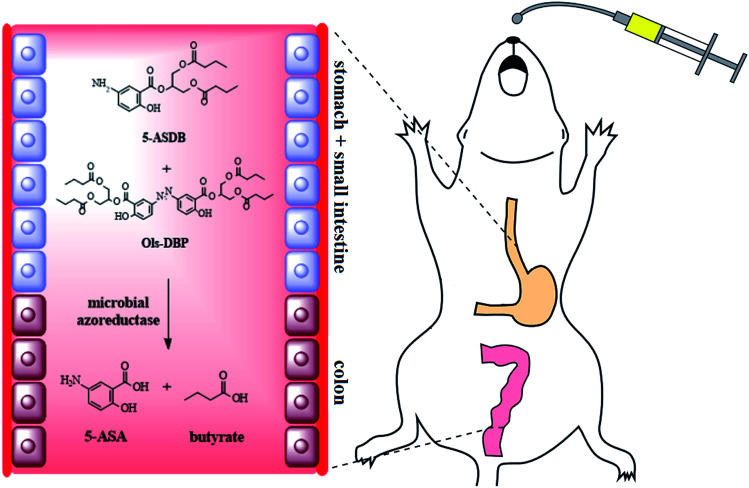
Four mutual prodrugs of 5-aminosalicylic acid and butyrate were designed and synthesized in this study. 5-ASDB and Ols-DBP were proved to have desirable colon-targeting properties and therapeutic effects on experimental colitis when orally administrated to mice.

In this study, four novel mutual prodrugs of 5-ASA and butyrate were designed and synthesized, their structural characterization, lipophilicity and aqueous solubility, *in vitro* and *in vivo* stability studies were also conducted in detail. Besides, the colon-targeting property and ameliorative effects of the synthesized prodrugs were evaluated by a series of indicators along with therapeutic comparison with a physical mixture of 5-ASA and butyrate on the experimental colitis in mice (Scheme 1).

## 2 Materials and methods

### 2.1 Materials

5-Aminosalicylic acid (5-ASA), 5-nitrosalicylic acid, butyric anhydride, olsalazine, 1,3-dihydroxyacetone, *N*-hydroxysuccinimide (NHS), *N*,*N*′-dicyclohexylcarbodiimide (DCC), *N*-(3-dimethylaminopropyl)-*N*′-ethylcarbodiimide hydrochloride (EDCI), 4-dimethylaminopyridine (DMAP) and palladium on activated charcoal (10% Pd/C) were purchased from Aladdin Bio-Chem Technology Co., Ltd (Shanghai, China). 2,4,6-Trinitrobenzenesulfonic acid (TNBS, 5%, w/v) and hexadecyltrimethylammonium bromide (HTAB) were purchased from Sigma-Aldrich Co., LLC (Shanghai, China). Myeloperoxidase (MPO), malondialdehyde (MDA), total superoxide dismutase (SOD), glutathione (GSH) and glutathione peroxidase (GSH-Px) kits were purchased from Jiancheng Bioengineering Institute (Nanjing, China).

Male Kunming mice (20–25 g) and male Sprague-Dawley rats (220–250 g) were obtained from Laboratory Animal Center of Xi'an Jiaotong University, SCXK (Shaanxi) 2007-001, China. Animals were housed under a 12 h light–dark cycle at a constant ambient temperature (22–25 °C), with normal chow and water *ad libitum*. They were allowed to acclimatize for 1 week before the experiments were started. All animal care and experimental protocols were in strict accordance with the Guidelines of the Laboratory Animal Center of Xi'an Jiaotong University and approved by the Institutional Animal Care and Use Committee of Xi'an Jiaotong University (No. XJTULAC2016-004).

### 2.2 Preparation of four mutual prodrugs of 5-aminosalicylic acid and butyrate

#### 2.2.1 Synthesis of 5-aminosalicylic acid butyrate (5-ASB)

5-Nitrosalicylic acid (4.6 g, 26 mmol) was mixed with butyric anhydride (6.4 mL, 39 mmol) in the presence of concentrated sulfuric acid (0.5 mL), the mixture was heated up to 189 °C and refluxed for 4 h. The product was dissolved in benzene and washed twice with water. The organic phase was collected, the solvent was evaporated in vacuum, and then a brown oil (5-nitrosalicylic acid butyrate) was obtained.^12^ The residual oil (2.0 g, 7.9 mmol) was dissolved in methanol (30 mL) and mixed with 10% Pd/C (300 mg), the mixture was reduced by hydrogen for 12 h. Finally, the solution was filtered, the methanol was evaporated in vacuum, and the residue was dissolved and placed onto a silica gel column, eluted with petroleum ether/ethyl acetate (2 : 1). The product was collected and evaporation of solvent in vacuum afforded a crystallized white solid (5-ASB).

#### 2.2.2 Synthesis of 2-(1,3-dibutyroxypropyl)-5-aminosalicylate (5-ASDB)

1,3-Dihydroxyacetone (1.5 g, 16.7 mmol) and butyric anhydride (10.91 mL, 67 mmol) were dissolved in anhydrous acetone (220 mL). Pyridine (10 mL) was dropwise added into the solution under nitrogen atmosphere with stirring at room temperature. The solvent was evaporated in vacuum, and residue was extracted with water/ethyl acetate. The organic phase was collected, washed with HCl (1 M) and NaHCO_3_ (1 M), dried with anhydrous sodium sulfate, and then a colorless oil was obtained. The oil residue was placed onto a silica gel column and eluted with petroleum ether/ethyl acetate (20 : 1). The product was collected and dried by vacuum evaporation, and then a crystallized white solid was obtained (1,3-dihydroxyacetone butyrate; 2.0 g, 58.3%).^13^

1,3-Dihydroxyacetone butyrate (1.0 g, 5.5 mmol) and NaBH_4_ (0.24 g) were dissolved in tetrahydrofuran (15 mL) and cooled to 5 °C. The reaction was terminated by glacial acetic acid (1 mL) 30 min later. The mixture was diluted with chloroform, washed with water and NaHCO_3_ (1 M), and dried with anhydrous magnesium sulfate, and then a colorless oil was obtained (1,3-glyceryl dibutyrate; 1.2 g, 94.5%).^14^

1,3-Glyceryl dibutyrate (2.0 g, 8.6 mmol), 5-nitrosalicylic acid (1.8 g, 10.32 mmol) and NHS (1.2 g, 10.30 mmol) were dissolved in anhydrous dichloromethane (50 mL), the mixture was stirred and cooled to 0 °C. After the ice bath was removed, DCC (4.4 g, 21.51 mmol), triethylamine (2.48 mL) and *N*,*N*′-dimethylformamide (0.5 mL) were added at room temperature and stirred for 30 h. The mixture was washed with HCl (1 M), saturated NaHCO_3_ and water, and dried with anhydrous magnesium sulfate. Residue was placed onto a silica gel column and eluted with petroleum ether/ethyl acetate (10 : 1). The product was collected and dried by vacuum evaporation, and then a crystallized white solid was obtained (2-(1,3-dibutyroxypropyl)-5-nitrosalicylate; 2.1 g, 60.4%).

2-(1,3-Dibutyroxypropyl)-5-nitrosalicylate (1.0 g, 2.5 mmol) and 10% Pd/C (100 mg) were dissolved in methanol (30 mL), the mixture was reduced by hydrogen for 7 h. The solution was filtered, the methanol was evaporated in vacuum, and finally, a crystallized yellow solid was obtained (5-ASDB).

#### 2.2.3 Synthesis of 3,3′-azobis(6-hydroxybenzoicacid)dibutyrate (Ols-DB)

Olsalazine (7.0 g, 23 mmol) was dissolved in butyric anhydride (38 mL, 230 mmol), the mixture was heated up to 81–82 °C and refluxed for 2 h. The reaction was monitored by a thin-layer chromatography method, with a developer of petroleum ether/ethyl acetate/trimethylamine (2 : 1 : 0.1). After the olsalazine was completely reacted, the mixture was washed with petroleum ether and filtered. Finally, a crystallized yellow solid was obtained (Ols-DB).

#### 2.2.4 Synthesis of 3,3′-azobis(2-(1,3-dibutyroxypropyl)-6-hydroxybenzoate) (Ols-DBP)

1,3-Glyceryl dibutyrate (1.3 g, 5.66 mmol), olsalazine (0.43 g, 1.42 mmol) and DMAP (0.13 g, 1.06 mmol) were dissolved in dichloromethane (30 mL), the mixture was cooled to 0 °C and stirred for 20 min. After the ice bath was removed, EDCI (1.6 g, 8.35 mmol; dissolved in 20 mL of dichloromethane) was added and stirred for 16 h at room temperature. The mixture was washed with water and saturated NaCl, and dried with anhydrous magnesium sulfate. Residue was placed onto a silica gel column and eluted with petroleum ether/ethyl acetate (10 : 1). The product was collected and dried by vacuum evaporation, and then a crystallized yellow solid was obtained (Ols-DBP).^15^

### 2.3 Characterization of 5-ASB, 5-ASDB, Ols-DB and Ols-DBP

The structure of the synthesized prodrugs were confirmed by differential scanning calorimetry (DSC), Fourier transform infrared spectroscopy (FTIR), ^1^H NMR and mass spectrometry (MS). The thermal behavior of each prodrug was analyzed by a differential scanning calorimeter (Mettler Toledo, DSC822e, Switzerland). Samples (5 mg) were placed in an aluminum crucible and heated at a scanning rate of 10 °C min^−1^ from 25 to 400 °C under nitrogen atmosphere. For FTIR analysis, samples and potassium bromide (1 : 100) were mixed uniformly and then analyzed by a FTIR-8400s infrared spectrophotometer (Shimadzu, Japan). The ^1^H NMR (in DMSO-d_6_) spectrum of each sample was recorded on a Varian 400 spectrometer (Varian, USA) at 400 MHz using tetramethylsilane (TMS) as internal standard. The MS analysis was performed by a GCMS-QP2010 mass spectrometer (Shimadzu, Japan) equipped with an electrospray ionization (ESI) system.

### 2.4 Analytical methods

The contents of 5-ASA, olsalazine and the synthesized prodrugs in experimental samples were determined by HPLC (Shimadzu, Japan) using a Diamonsil ODS-2 C18 column. The detecting conditions were as follows: (1) 5-ASB: the mobile phase was 55% of methanol solution containing 0.5% acetic acid, the wavelength for UV detector was 335 nm; (2) olsalazine: the mobile phase was a mixture of methanol/phosphate buffer (pH 7.4, 60 : 40), the wavelength was 360 nm; (3) 5-ASDB: the mobile phase was 40% of methanol solution, the wavelength was 353 nm; (4) Ols-DB: the mobile phase was 85% of methanol solution containing 0.5% acetic acid, the wavelength was 328 nm. (5) Ols-DBP: the mobile phase was 90% of methanol solution, the wavelength was 335 nm; (6) 5-ASA: the mobile phase was a mixture of methanol/phosphate buffer (pH 6.5, 15 : 85), the wavelength was 330 nm. Samples were detected at room temperature, the flow rate was 1.0 mL min^−1^.

The linearity of concentrations for each compound was assessed by a calibration that obtained by plotting the peak area of each sample *versus* its concentration, respectively. The absolute recoveries of each compound from different samples were calculated by analyzing spiked samples at 1.0, 5.0 and 20.0 μg mL^−1^, respectively, and each concentration level was tested 5 times. For precision evaluation, samples (1.0, 5.0 and 20.0 μg mL^−1^) were analyzed 5 times within 1 day (intra-day) and within 5 days (inter-day) for calculating the relative standard deviation (RSD), respectively.

### 2.5 Determination of lipophilicity parameters and aqueous solubility

The partition coefficients of 5-ASA and the synthesized prodrugs were determined in *n*-octanol/phosphate buffer (pH 7.4) systems. The phosphate buffer and octanol were mutually saturated at 20–22 °C before use. Samples were dissolved in the aqueous phase and the *n*-octanol/phosphate buffer mixtures were shaken for 24 h to reach a distribution equilibrium. The volumes of each phase were chosen so that the solute concentration in the aqueous phase, before and after distribution, could readily be measured using HPLC method. The partition coefficients (*P*) were calculated from the following equation:
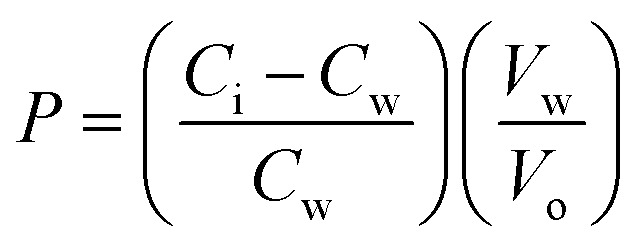
where *C*_i_ and *C*_w_ represent the solute concentrations in the aqueous buffer phase before and after distribution, respectively, *V*_w_ represents the volume of the aqueous phase, and *V*_o_ is the volume of the octanol phase.^16^

The solubility (*S*) of the prodrugs in water was determined at 25 ± 2 °C by adding excess amounts of the compounds to distilled water in tubes. The mixtures were placed in an ultrasonic water bath for 10 min and then shaken for 24 h to establish a saturation equilibrium. Upon filtration, the filtrate was diluted with an appropriate amount of water and the mixture was analyzed by HPLC, the concentration of each compound in its saturated solution was calculated.

### 2.6 *In vitro* and *in vivo* stability studies

#### 2.6.1 *In vitro* incubation with stomach and small intestine contents of rats

After anesthetized with diethyl ether, rats were sacrificed and their contents of stomach and small intestine were collected and homogenized respectively, the homogenate was diluted with buffer solution (pH 1.2 for stomach contents, pH 6.8 for small intestine contents) to 20% (w/v). Then 1 mL of prodrug solution was added to 5 mL of above diluent (equivalent to 100 μg mL^−1^ of 5-ASA) and co-incubated at 37 °C for 4 h. After 0.25, 0.5, 1.0, 1.5, 2.0, 3.0 and 4.0 h of incubation, 200 μL of samples were collected and replaced with the same volume of blank homogenate, then samples were centrifuged at 13 000 rpm for 5 min, filtered with membrane filter, and analyzed by HPLC. The residual contents of each prodrug in homogenate were calculated and the hydrolysis curves were drawn.

#### 2.6.2 *In vivo* incubation in the colon of rats

SD rats were anesthetized and cut through the linea alba, and 1 mL of prodrug solution (equivalent to 100 μg mL^−1^ of 5-ASA, dissolved in phosphate buffer, pH 7.4) was injected into their colons, then both ends of the colon were tied and their abdomen was sutured.^17^ 6 hours later, rats were sacrificed and their colon contents were separated and homogenized in 40 mL of phosphate buffer (pH 7.4). The homogenate was sonicated for 5 min and centrifuged at 13 000 rpm for 5 min, then 0.2 mL of the supernatant was collected and mixed with 0.8 mL of methanol. Finally, samples were centrifuged, filtered and analyzed by HPLC. The residual contents of each prodrug in colon contents were calculated, and the hydrolysis ratio was calculated by the following equation:
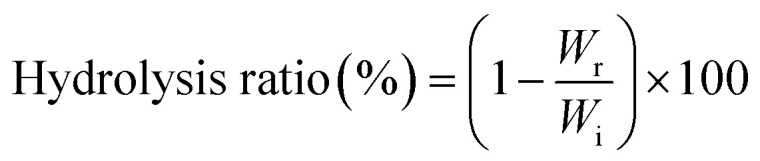
where *W*_r_ is the residual amount of prodrugs in the colon contents and *W*_i_ is the initial amount of prodrugs that administered to rats.

### 2.7 Ameliorative effects of prodrugs on experimental colitis

#### 2.7.1 Colitis induction and experiment protocols

Colitis was induced according to the previous study.^18,19^ Briefly, after 12 h of fasting, mice were anesthetized with intraperitoneal pentobarbital sodium (35 mg kg^−1^) before the induction of colitis. 0.1 mL of 50% (v/v) ethanol contained 2.5% (v/v) TNBS was instilled into the colon 3.5–4 cm from the anus by a gavage needle. The mice were kept in a head-down position for 30 s to prevent leakage of the intracolonic instillation. Mice in the control group received normal saline instead of TNBS solution, a physical mixture of 5-ASA and sodium butyrate was used as the positive control. Mice in test groups received different amount of prodrugs at an equivalent dose of 100 mg kg^−1^ 5-ASA.^20,21^ 84 mice were randomly divided into 7 groups (*n* = 12): (1) control – no colitis induced (i.g., saline), (2) TNBS (i.g., saline), (3) positive (i.g., 100 mg kg^−1^ 5-ASA + 40 mg kg^−1^ sodium butyrate), (4) 5-ASB (i.g., 146 mg kg^−1^), (5) 5-ASDB (i.g., 240 mg kg^−1^), (6) Ols-DB (enem., 140 mg kg^−1^), (7) Ols-DBP (i.g., 240 mg kg^−1^). The treatments were given once daily for 5 continuous days starting 24 hours after the induction of colitis.

#### 2.7.2 Body weight and survival rates of mice

After the induction of colitis, the body weight of each mice was recorded daily, and the curves of weight changes for each group were drawn to evaluate the TNBS-induced colitis. Meanwhile, the death of mice in each group was recorded daily and the final survival rates of mice were calculated.

#### 2.7.3 Measurement of DAI score

The disease activity index (DAI) of mice was recorded and scored daily, the scoring criteria are shown in Table 1. DAI is ranged from 0 to 4 and it is the sum of scores given for body weight loss, stool consistency and presence or absence of fecal blood. The fecal occult blood of mice was evaluated by the *ο*-tolidine method.^22^ A small amount of feces samples were smeared on a white plate. 2–3 drops of *ο*-tolidine solution (10 g L^−1^, dissolved in acetic acid) were added into samples and mixed evenly. Then 2–3 drops of 3% H_2_O_2_ solution were added into the mixture, meanwhile, start the time and observe immediately. Scored as: normal, weak positive, green to light green developing over 2 min; +, positive, greenish-blue to deep green developing within 1 min; ++, positive, blue to bluish-green developing within 5–20 s; +++, very strong positive, intense blue color developing immediately.^23^

**Table 1:** The evaluation criteria of disease activity index (DAI) of mice

Weight loss	Stool consistency	Fecal occult blood	Score
None	Well-formed pellets	Normal	0
1–5%	Loose stools	Occult blood +	1
5–10%		Occult blood ++	2
10–15%	Diarrhea	Occult blood +++	3
>15%		Visible gross bleeding	4

#### 2.7.4 Measurement of colon index and CDS

At the end of the experiment, all mice were sacrificed and their entire colons were excised, opened longitudinally and washed carefully with normal saline. After dried with filter papers, colons were weighted and the length of colons was measured and recorded. The colon index was calculated as follows:Colon index (g dm^−1^) = weight of colon/length of colon

The colonic damage score (CDS) of mice in each group was evaluated according to the following criteria: 0, no ulcer and colitis; 1, no ulcer, local congestion; 2, ulcers, no local congestion; 3, ulcer and colitis at only one site; 4, ulcer and colitis at more than one sites; 5, over 2 cm of ulcer surface.^24^

#### 2.7.5 Measurement of MPO activity

Clean and dry colon tissue samples were suspended in 0.5% HTAB in ice-cold potassium phosphate buffer (pH 7.4, 50 mmol L^−1^), the mixture (50 g L^−1^) was homogenized at 4 °C for 30 s and then sonicated in an ice bath for 10 s. Samples were freeze-thawed three times, after which sonication was repeated. Homogenates were then centrifuged at 13 000 rpm for 30 min and MPO was extracted from the resulting supernatant.^25^ MPO activity was measured by the assay kit according to its provider's instructions.

#### 2.7.6 Levels of SOD, MDA, GSH and GSH-Px

Colon tissue samples were homogenized with normal saline, the homogenate (10%, w/w) was centrifuged at 13 000 rpm for 15 min, and the resulting supernatant was diluted to 1% (w/w) for further assay. The protein levels of the colon homogenate were detected by a BCA protein assay kit. Then the activities of SOD and GSH-Px, and the levels of MDA and GSH of samples in each group were measured by the assay kits according to the provider's instructions.

### 2.8 Statistical analysis

Statistical analyses were performed with SPSS version 13.0 for Windows. One-way ANOVA followed by the least significant difference (LSD) test were used to test the difference between the data. Results were expressed as mean ± SD. *P* < 0.05 was considered to be statistically significant.

## 3 Results

### 3.1 Preparation and characterization of 5-ASB, 5-ASDB, Ols-DB and Ols-DBP

**Fig. 1:**
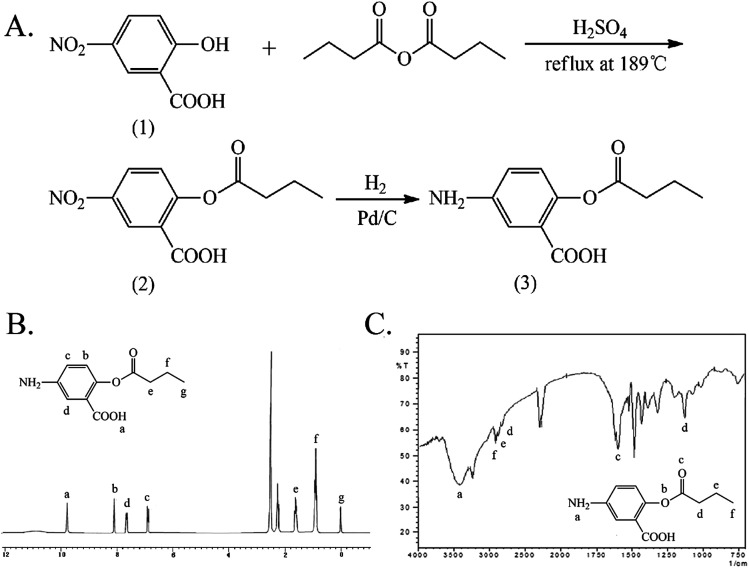
The synthesis and characterization of 5-ASB. (A) The synthetic route of 5-ASB. (B) The ^1^H NMR spectrum of 5-ASB. (C) The FTIR spectrum of 5-ASB. 5-ASB, 5-aminosalicylic acid butyrate.

The synthesis and characterization of 5-ASB are shown in Fig. 1. The synthetic route is similar to that of aspirin, the hydroxyl of 5-nitrosalicylic acid (1) was reacted with the carboxyl groups of butyric anhydride under the catalysis of concentrated sulfuric acid to obtain the 5-nitrosalicylic acid butyrate (2). Then the nitro group of (2) was reduced by the Pd/C and hydrogen and the final product 5-ASB (3) was obtained. Yield: 1.11 g, 63.3%. mp: 214–216 °C. IR (KBr), *ν*_max_ (cm^−1^): 3475 (–NH_2_), 2962 (–CH_3_), 2929 (–CH_2_–), 2875 (–CH_2_–), 1650 (C=O), 1189 (C–O–C). ^1^H NMR (DMSO-d_6_): *δ* 0.91 (t, 3H, αC), 1.61 (m, 2H, βC), 2.22 (t, 2H, γC), 6.91 (d, 1H, ArH), 7.67 (d, 1H, ArH), 8.11 (s, 1H, ArH), 9.80 (s, 1H, –COOH). ESI-MS *m*/*z* 223 [M + H]^+^.

**Fig. 2:**
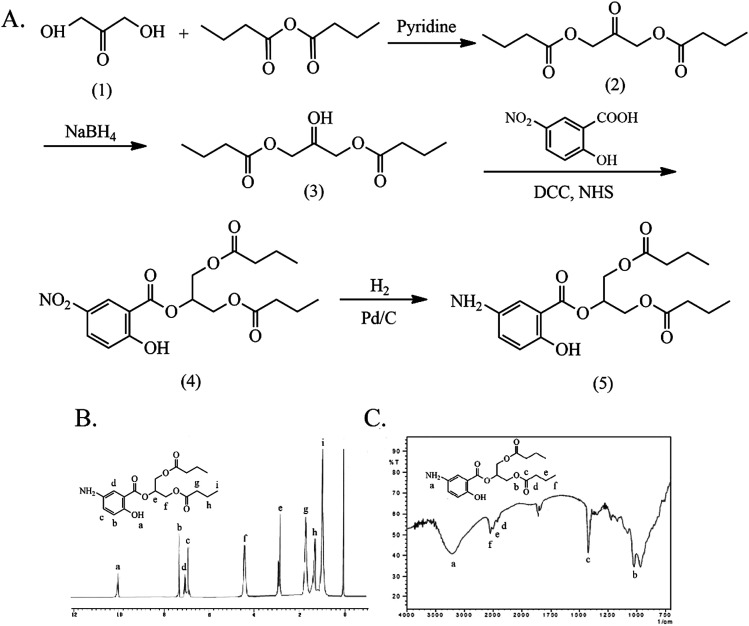
The synthesis and characterization of 5-ASDB. (A) The synthetic route of 5-ASDB. (B) The ^1^H NMR spectrum of 5-ASDB. (C) The FTIR spectrum of 5-ASDB. 5-ASDB, 2-(1,3-dibutyroxypropyl)-5-aminosalicylate.

The synthesis and characterization of 5-ASDB are shown in Fig. 2. The esterification was reacted between the hydroxyls of 1,3-dihydroxyacetone (1) and the carboxyl groups of butyric anhydride under the catalysis of pyridine to obtain the 1,3-dihydroxyacetone butyrate (2). The carbonyl group of (2) was reduced by NaBH_4_ to obtain the 1,3-glyceryl dibutyrate (3), and then the (3) was reacted with 5-nitrosalicylic acid under the catalysis of DCC/NHS to produce the 2-(1,3-dibutyroxypropyl)-5-nitrosalicylate (4). Finally, the nitro group of (4) was reduced by the Pd/C and hydrogen, and the final product 5-ASDB (5) was obtained. Yield: 0.88 g, 96%. mp: 250–252 °C. IR (KBr), *ν*_max_ (cm^−1^): 3456 (–NH_2_), 2962 (–CH_3_), 2939 (–CH_2_–), 2877 (–CH_2_–), 1736 (C=O), 1173 (C–O–C), 856 (–Ar), 1011 (–Ar). ^1^H NMR (DMSO-d_6_): *δ* 0.93 (t, 6H, αC), 1.63 (m, 4H, βC), 2.31 (t, 4H, γC), 6.88 (d, 1H, ArH), 7.01 (d, 1H, ArH), 7.26 (s, 1H, ArH), 9.99 (s, 1H, –ArOH). ESI-MS *m*/*z* 367 [M + H]^+^.

**Fig. 3:**
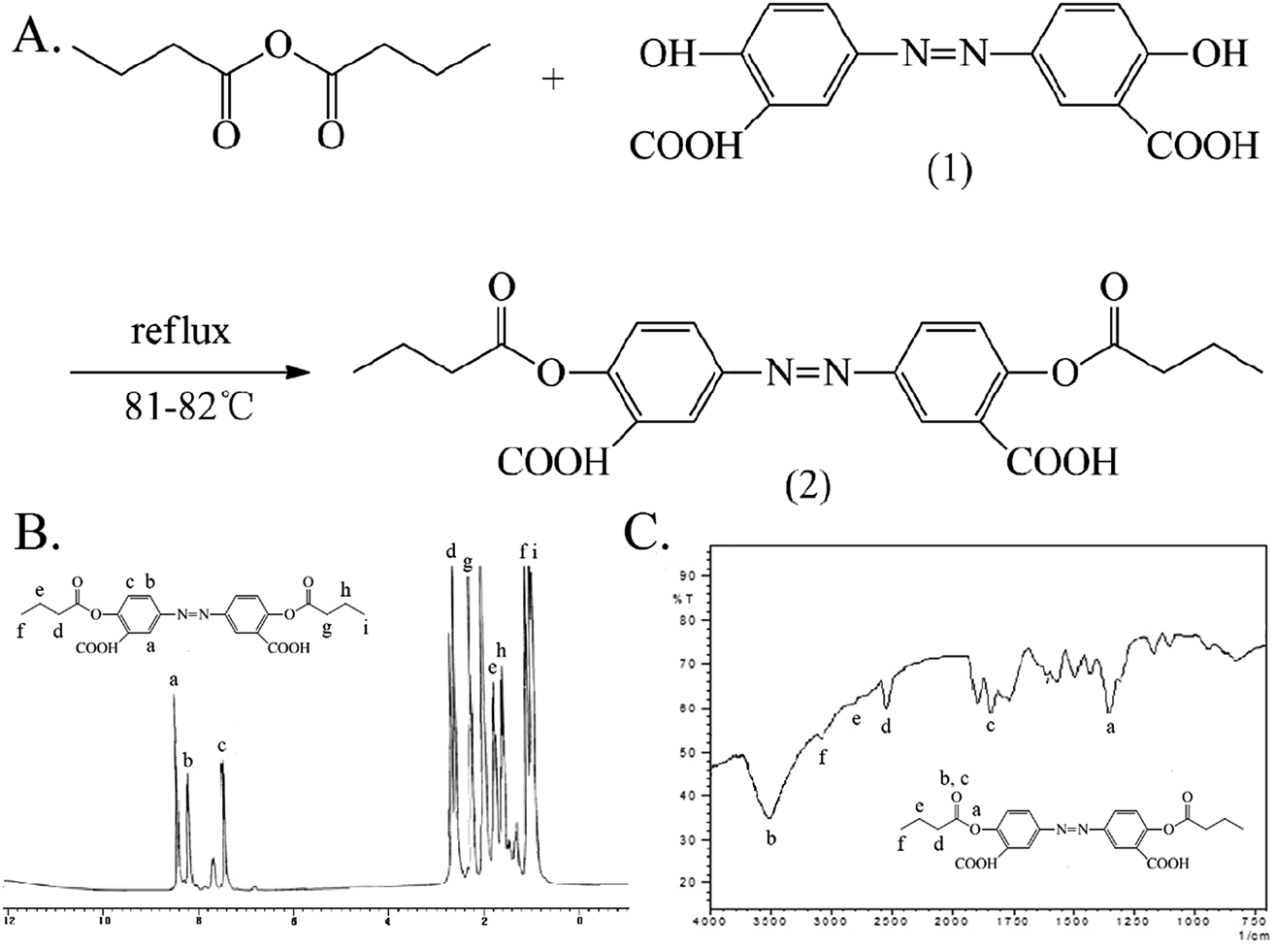
The synthesis and characterization of Ols-DB. (A) The synthetic route of Ols-DB. (B) The ^1^H NMR spectrum of Ols-DB. (C) The FTIR spectrum of Ols-DB. Ols-DB, 3,3′-azobis(6-hydroxybenzoicacid)dibutyrate.

The synthesis of Ols-DB is similar to that of aspirin and the characterization results are shown in Fig. 3. The hydroxyls of olsalazine (1) were reacted with the carboxyl groups of butyric anhydride *via* esterification, the Ols-DB (2) was obtained under reflux conditions. The synthetic route is simple and with convenient disposition. Yield: 9.0 g, 95.7%. mp: 216–218 °C. IR (KBr), *ν*_max_ (cm^−1^): 2974 (–CH_3_), 2665 (–CH_2_–), 2353 (–CH_2_–), 3458 (C=O), 1650 (C=O), 1144 (C–O–C). ^1^H NMR (DMSO-d_6_): *δ* 0.88 (t, 3H, αC), 1.00 (t, 3H, αC), 1.53 (m, 2H, βC), 1.68 (m, 2H, βC), 2.16 (t, 2H, γC), 2.60 (t, 2H, γC), 7.47 (d, 2H, ArH), 8.21 (d, 2H, ArH), 8.43 (s, 2H, ArH). ESI-MS *m*/*z* 443.

**Fig. 4:**
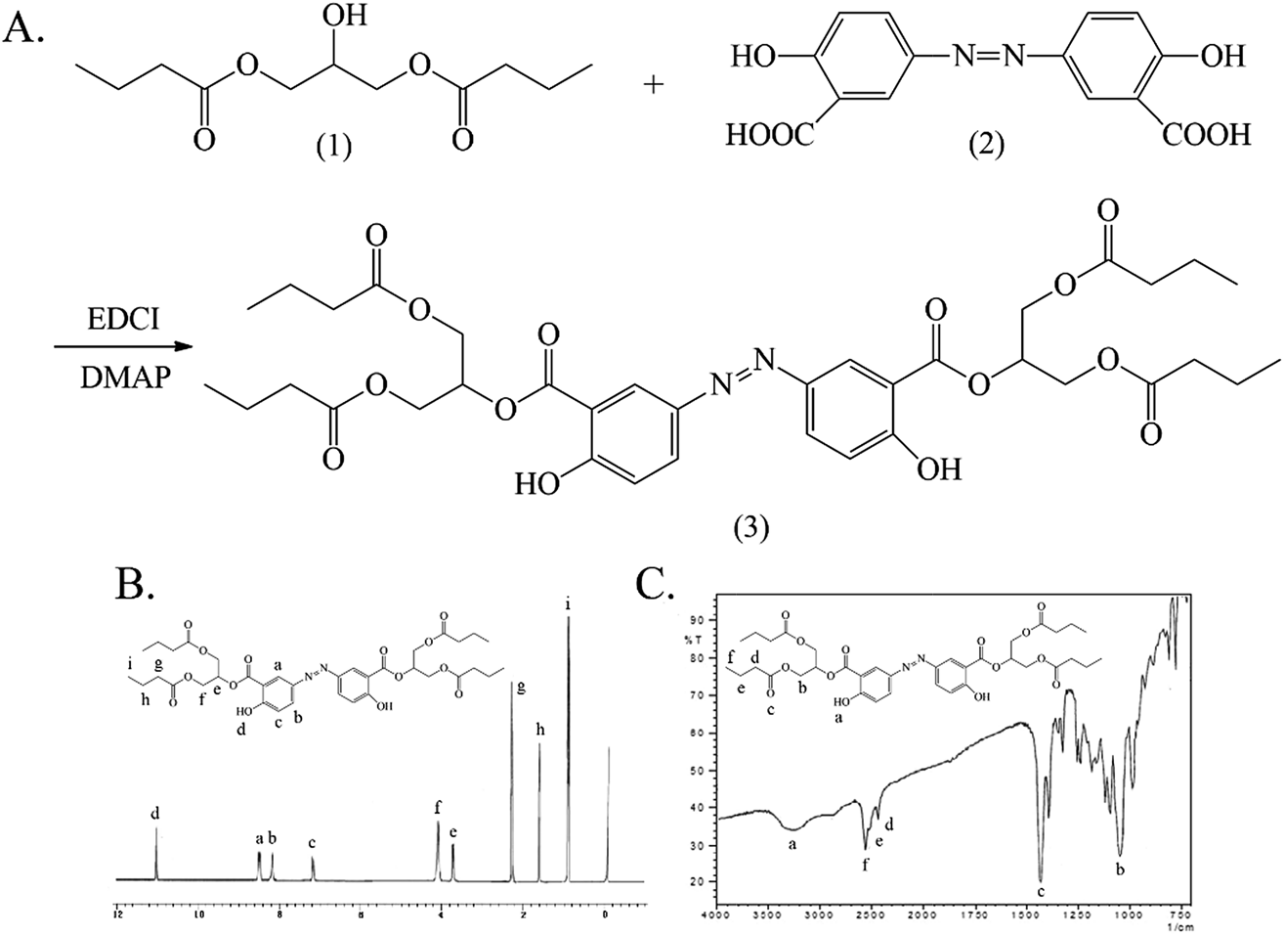
The synthesis and characterization of Ols-DBP. (A) The synthetic route of Ols-DBP. (B) The ^1^H NMR spectrum of Ols-DBP. (C) The FTIR spectrum of Ols-DBP. Ols-DBP, 3,3′-azobis(2-(1,3-dibutyroxypropyl))-6-hydroxybenzoate.

The synthesis and characterization of Ols-DBP are shown in Fig. 4. The hydroxyls of 1,3-glyceryl dibutyrate (1) were reacted with the carboxyl groups of olsalazine (2) under the catalysis of EDCI/DMAP, the Ols-DBP (3) was obtained *via* esterification. Yield: 0.51 g, 49.5%. mp: 234–235 °C. IR (KBr), *ν*_max_ (cm^−1^): 3454 (–ArOH), 2970 (–CH_3_), 2930 (–CH_2_–), 2878 (–CH_2_–), 1736 (C=O), 1180 (C–O–C), 841 (–Ar), 861 (–Ar). ^1^H NMR (DMSO-d_6_): *δ* 0.88 (t, 12H, αC), 1.56 (m, 8H, βC), 2.27 (t, 8H, γC), 7.20 (d, 2H, ArH), 8.05 (d, 2H, ArH), 8.24 (s, 2H, ArH), 10.85 (s, 1H, –ArOH), 10.88 (s, 1H, –ArOH). ESI-MS *m*/*z* 729.

### 3.2 Analytical method

A linear relationship was established between the concentration (0.4–40 μg mL^−1^) and the peak area of each sample. The regression equations of 5-ASA, olsalazine, 5-ASB, 5-ASDB, Ols-DB and Ols-DBP in different tissue samples for HPLC analysis are listed in Table 2. The absolute recoveries of the above six compounds at each concentration (1.0, 5.0 and 20.0 μg mL^−1^) were >90%. The recoveries had no significant difference between the three concentrations. For precision assay, both the intra- and inter-day RSD in all tissue samples were <6%. These data validated that the HPLC method were acceptable and reproducible (Table 3).

**Table 2:** Linear equations of 5-ASA, olsalazine, 5-ASB, 5-ASDB, Ols-DB and Ols-DBP in different tissue samples for HPLC analysis

Samples		Equations	*R* ^2^	LOQ (μg mL^−1^)
5-ASA	Phosphate buffer (pH 7.4)	*y* = 34 727*x* − 4623	0.9998	0.4
	Stomach contents	*y* = 26 525*x* − 5728	0.9995	0.4
	Small intestine contents	*y* = 27 473*x* − 2294	0.9993	0.4
	Colon contents	*y* = 27 325*x* − 3865	0.9990	0.4
Olsalazine	Phosphate buffer (pH 7.4)	*y* = 50 283*x* + 2702	0.9987	0.4
	Stomach contents	*y* = 79 820*x* + 1610	0.9991	0.4
	Small intestine contents	*y* = 79 200*x* + 1940	0.9993	0.4
	Colon contents	*y* = 27 325*x* − 8865	0.9990	0.4
5-ASB	Phosphate buffer (pH 7.4)	*y* = 14 948*x* − 1093	0.9995	0.4
	Stomach contents	*y* = 12 636*x* + 1704	0.9996	0.4
	Small intestine contents	*y* = 10 203*x* + 1832	0.9988	0.4
	Colon contents	*y* = 12 129*x* + 1385	0.9997	0.4
5-ASDB	Phosphate buffer (pH 7.4)	*y* = 16 977*x* − 4623	0.9994	0.4
Ols-DB		*y* = 18 538*x* + 6421	0.9978	0.4
Ols-DBP		*y* = 20 218*x* − 5753	0.9998	0.4

**Table 3:** Recovery and precision for the detection of 5-ASA, olsalazine, 5-ASB, 5-ASDB, Ols-DB and Ols-DBP in different tissue samples by HPLC (data are means ± SD., *n* = 5)

Samples		Concentration (μg mL^−1^)	Recovery (%)	Intra-day RSD (%)	Inter-day RSD (%)
5-ASA	Phosphate buffer (pH 7.4)	1.0	97.0 ± 3.0	3.5	4.0
		5.0	105.0 ± 1.3	3.5	2.8
		20.0	98.5 ± 3.0	2.0	2.5
	Stomach contents	1.0	112.0 ± 2.1	4.8	4.5
		5.0	97.0 ± 2.8	3.0	3.6
		20.0	97.0 ± 2.8	2.8	2.9
	Small intestine contents	1.0	98.0 ± 2.6	2.6	2.7
		5.0	104.3 ± 3.8	3.8	4.2
		20.0	97.0 ± 1.7	1.7	2.5
	Colon contents	1.0	111.3 ± 2.9	3.9	5.0
		5.0	96.6 ± 2.7	3.0	3.0
		20.0	96.2 ± 2.3	2.3	3.2
Olsalazine	Phosphate buffer (pH 7.4)	1.0	103.3 ± 2.5	3.1	2.7
		5.0	97.4 ± 3.3	2.5	2.8
		20.0	98.3 ± 1.5	2.0	3.1
	Stomach contents	1.0	97.9 ± 5.0	5.0	5.5
		5.0	99.5 ± 2.8	2.8	3.6
		20.0	98.3 ± 2.2	2.1	3.9
	Small intestine contents	1.0	94.4 ± 2.4	2.4	4.6
		5.0	103.7 ± 2.8	2.7	3.5
		20.0	101.4 ± 1.7	1.7	2.9
	Colon contents	1.0	111.3 ± 2.9	3.9	5.2
		5.0	96.6 ± 2.7	3.0	3.0
		20.0	96.2 ± 2.3	2.3	3.2
5-ASB	Phosphate buffer (pH 7.4)	1.0	96.8 ± 3.5	2.5	2.3
		5.0	98.5 ± 2.0	2.2	2.0
		20.0	100.8 ± 2.7	1.7	1.5
	Stomach contents	1.0	113.4 ± 2.0	2.2	2.2
		5.0	94.6 ± 1.0	1.0	1.2
		20.0	101.9 ± 1.2	1.2	1.3
	Small intestine contents	1.0	93.8 ± 3.6	3.0	2.6
		5.0	100.5 ± 1.4	1.5	1.5
		20.0	105.4 ± 1.1	1.1	1.8
	Colon contents	1.0	97.5 ± 2.0	1.8	2.0
		5.0	96.6 ± 1.7	1.0	1.3
		20.0	102.8 ± 3.1	3.2	3.5
5-ASDB	Phosphate buffer (pH 7.4)	1.0	102.2 ± 2.3	2.7	3.3
		5.0	98.6 ± 2.5	2.6	2.8
		20.0	101.4 ± 1.9	1.7	2.0
Ols-DB		1.0	95.5 ± 3.7	3.7	4.2
		5.0	96.1 ± 4.2	2.6	4.5
		20.0	100.9 ± 3.6	2.2	3.1
Ols-DBP		1.0	101.8 ± 2.2	3.3	4.7
		5.0	99.4 ± 2.0	4.0	3.2
		20.0	100.5 ± 4.2	2.2	3.0

### 3.3 Lipophilicity and solubility of prodrugs

The lipophilicity and solubility of each compound were determined to evaluate their potential as colon-targeting prodrugs. As shown in Table 4, the enhanced aqueous solubility of all the prodrugs (0.08 ± 0.02 to 0.31 ± 0.05 g mL^−1^) and decreased partition coefficients in *n*-octanol/phosphate buffer (pH 7.4) (0.16 ± 0.03 to 0.58 ± 0.04) as compared to 5-ASA (*S* = 1.31 ± 0.2 mg mL^−1^, log *P* = 0.65 ± 0.02) suggested that their absorption from the upper gastrointestinal tract would be minimum thus facilitating the passage of prodrugs directly to colon.

**Table 4:** Partition coefficients (log *P*) and aqueous solubility (*S*) of 5-ASA, 5-ASB, 5-ASDB, Ols-DB and Ols-DBP (data are means ± SD, *n* = 3)

Samples	*P*	log *P*	*S* (g mL^−1^)
5-ASA	4.47 ± 0.21	0.65 ± 0.02	0.0013 ± 0.0002
5-ASB	1.45 ± 0.10	0.16 ± 0.03	0.31 ± 0.05
5-ASDB	3.81 ± 0.35	0.58 ± 0.04	0.08 ± 0.02
Ols-DB	2.10 ± 0.24	0.32 ± 0.05	0.27 ± 0.03
Ols-DBP	2.94 ± 0.37	0.47 ± 0.06	0.19 ± 0.04

### 3.4 *In vitro* and *in vivo* stability study of prodrugs

**Fig. 5:**
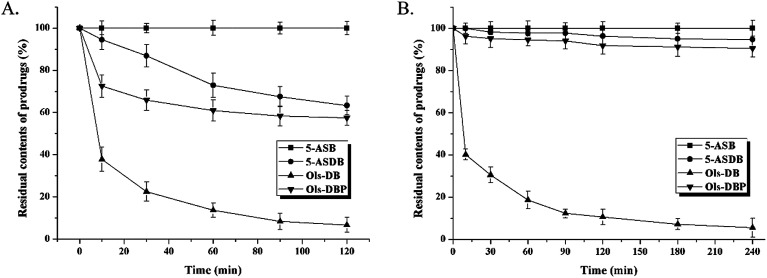
The *in vitro* hydrolysis behavior of 5-ASB, 5-ASDB, Ols-DB and Ols-DBP in the environment of upper gastrointestinal tract. (A) Prodrugs were incubated in the stomach contents of rats for 2 hours, the residual contents of each prodrugs were calculated at predetermined intervals. (B) Prodrugs were incubated in the small intestine contents of rats for 4 hours, the residual contents of each prodrug were calculated at predetermined intervals. Data are shown as mean ± SD, *n* = 5.

The hydrolysis behaviors of 5-ASB, 5-ASDB, Ols-DB and Ols-DBP in stomach contents and small intestine contents of rats are shown in Fig. 5. The residual contents of each prodrug in stomach contents are shown in Fig. 5A. After 2 h of incubation, 5-ASB was extremely stable in stomach and hardly hydrolyzed, on the contrary, Ols-DB was highly unstable and had been rapidly and almost completely decomposed. 5-ASDB and Ols-DBP were gradually hydrolyzed in the acid environment of stomach, 63.3% of 5-ASDB and 57.5% of Ols-DBP were left after 2 h of incubation. As shown in Fig. 5B, 5-ASB, 5-ASDB and Ols-DBP were all relatively stable in the environment of small intestine, their residual contents were 100%, 94.7% and 90.4%, respectively. However, only 5.5% of Ols-DB was left in the small intestine contents after 4 h of incubation.

**Fig. 6:**
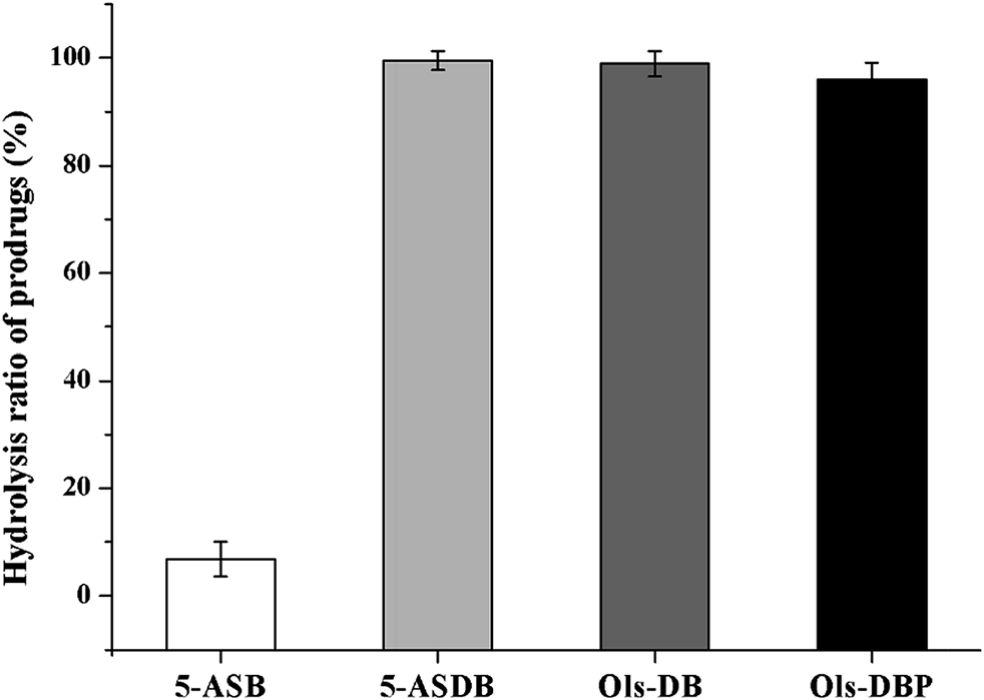
The hydrolysis ratio of 5-ASB, 5-ASDB, Ols-DB and Ols-DBP after incubated in rat colons. After 6 hours of incubation, prodrugs were hydrolyzed in the colon environment. The hydrolysis ratio of each prodrug was calculated to evaluate their hydrolysis behavior in the colon and their colon-targeting properties. Data are shown as mean ± SD, *n* = 5.

The hydrolysis ratios of each prodrug after *in vivo* incubation are shown in Fig. 6. After incubated in rat colons for 6 h, 5-ASDB, Ols-DB and Ols-DBP were almost hydrolyzed completely. However, 5-ASB was still hardly hydrolyzed (6.79%), which may seem to be inconsistent with the initial expectation of the colon-targeting prodrugs, and its therapeutic effects should be further confirmed by pharmacodynamics studies.

### 3.5 Effects of prodrugs on survival rates and body weight of mice

**Fig. 7:**
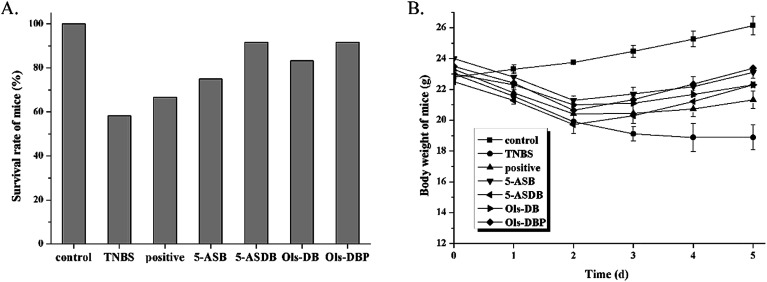
Effects of prodrugs on survival rates and body weight of mice. (A) Mice in each group were administrated with different drugs (equivalent to 100 mg kg^−1^ of 5-ASA) for 5 days, the survival rates of mice in each group were calculated at the end of the experiment. (B) The body weight of mice in each group was recorded daily. Data are shown as mean ± SD, *n* = 12.

Survival rates and body weight were used to evaluate the effects of prodrugs on mice (Fig. 7). As shown in Fig. 7A, expect for the control group, the death of mice were found in all the test groups during the experiment, especially in the TNBS group (58.3%). But the survival rates showed little difference among the test groups, which may due to the small numbers of animals (*n* = 12). As shown in Fig. 7B, the body weight of mice in the control group was increased steadily, while the TNBS group showed a continuous downward trend. The positive, 5-ASDB, Ols-DB and Ols-DBP group all presented a slight weight loss at first and gained gradually from the 2^nd^ day. These results suggested that the colitis models were established successfully, 5-ASB, 5-ASDB, Ols-DB and Ols-DBP all had certain therapeutic effects on the TNBS-induced UC.

### 3.6 Effects of prodrugs on DAI of mice

**Fig. 8:**
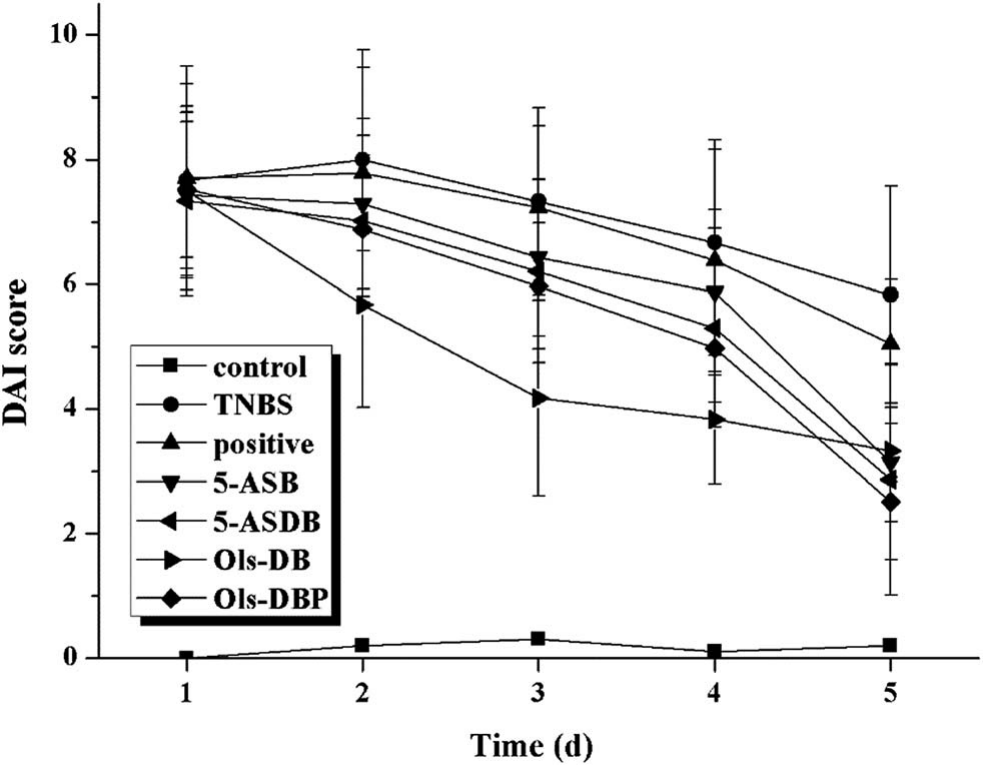
Effects of prodrugs on DAI of mice. After the induction of colitis, mice in each group were administered with different drugs (equivalent to 100 mg kg^−1^ of 5-ASA). The DAI of mice was recorded and scored daily to evaluate the potential therapeutic effects of prodrugs. DAI, disease activity index. Data are shown as mean ± SD, *n* = 12.

In mice with TNBS-induced experimental colitis, we observed bloody stools, diarrhea, reducing diet, hypokinesia, and with different degrees of huddle and weight loss, which ultimately resulted in a sharp increase of the DAI at day 1, compared with the control group (Fig. 8). Different compounds were administered once daily for 5 days at the same dose (equivalent to 100 mg kg^−1^ of 5-ASA) to detect potential therapeutic effects. After treatment, the DAI of test groups was significantly reduced from day 3, besides, 5-ASB, 5-ASDB, Ols-DB and Ols-DBP had better effects than the positive control.

### 3.7 Effects of prodrugs on colon index and CDS of mice

**Fig. 9:**
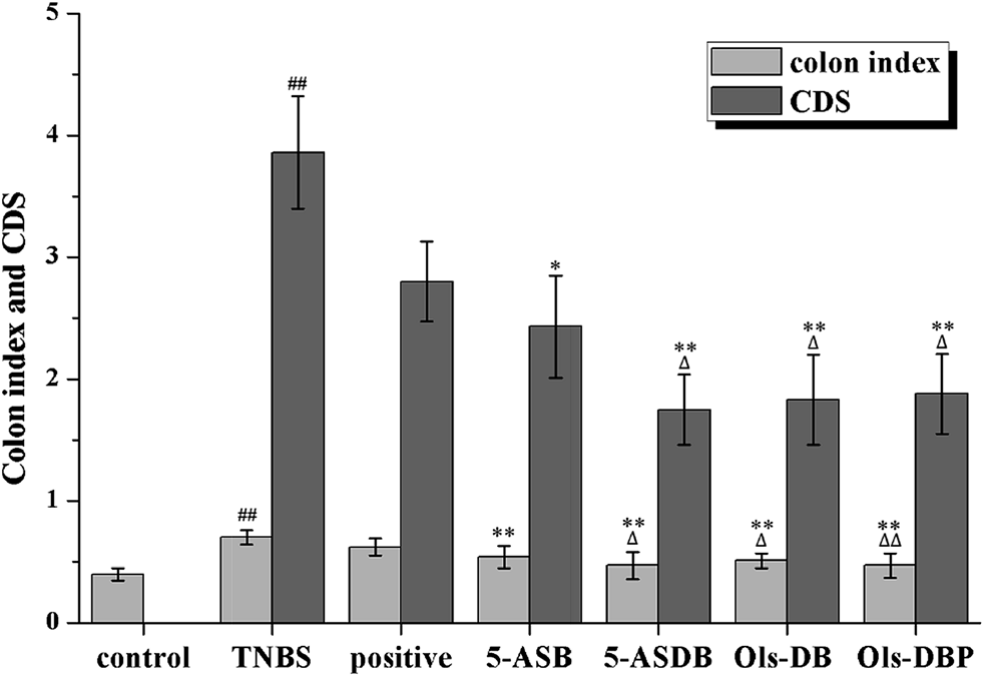
Effects of prodrugs on colon index and CDS of mice. (A) At the end of the experiment, all mice were sacrificed and their entire colons were excised and measured to calculate the colon index. (B) The CDS of mice in each group was scored according to the macroscopical changes of the colon. CDS, colonic damage score. ^##^: *p* < 0.01 *vs.* control group; *: *p* < 0.05, **: *p* < 0.01 *vs.* TNBS group; △: *p* < 0.05, △△: *p* < 0.01 *vs.* positive control group. Data are shown as mean ± SD, *n* = 12.

The experimental colitis would induce compensatory hypertrophy in the colon of mice, cause colon wall thickening and colon length shortening, which eventually leaded to an increase of the colon index. As shown in Fig. 9, the colon index of TNBS group was significantly higher than that of the control group (*p* < 0.01), suggested that the mouse models were successfully established. The colon indexes of mice in test groups were all significantly decreased after treatment (*p* < 0.01), as compared with the TNBS group. Besides, 5-ASDB (*p* < 0.05) and Ols-DBP (*p* < 0.01) were significantly better than the positive control. Mice treated with Ols-DBP showed a similar colon index to that of the control group.

After mice were dissected, results showed that the inflammatory lesions involved the total colons of the TNBS-treated mice, mainly manifested as edema, transmural injury and necrosis, and deformation of the colon. The colonic mucosa damage of mice was evaluated and scored (Fig. 9). The CDS of the TNBS group was significantly higher than that of the control group (*p* < 0.01). Compared with the TNBS group, the CDSs of all the teat groups were obviously decreased. 5-ASDB, Ols-DB and Ols-DBP were significantly greater than the positive control (*p* < 0.05).

### 3.8 Effects of prodrugs on MPO activity of mice

**Fig. 10:**
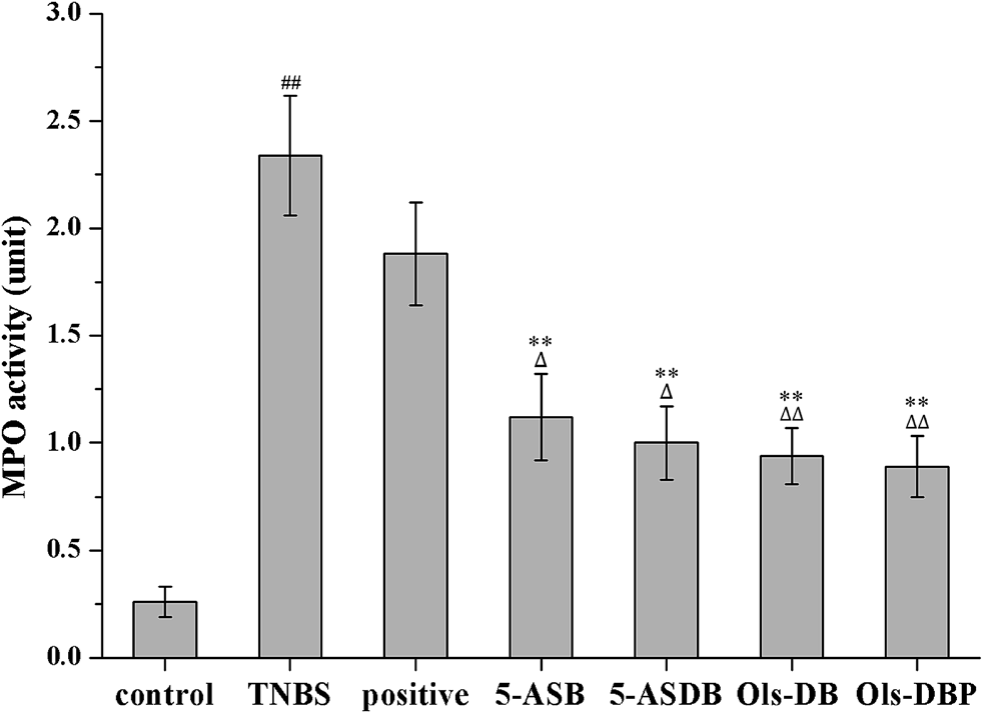
Effects of prodrugs on MPO activity of mice. After treatment, the MPO activities of mice in colonic tissues were measured to evaluate the potential therapeutic effects of prodrugs. MPO, myeloperoxidase. ^##^: *p* < 0.01 *vs.* control group; **: *p* < 0.01 *vs.* TNBS group; △: *p* < 0.05, △: *p* < 0.01 *vs.* positive control group. Data are shown as mean ± SD, *n* = 12.

MPO is an important peroxidases and it is unique to neutrophils and monocytes, which exists mainly in azurophilic granules. In this study, MPO is used as a quantitative indicator of inflammation due to the correlation between MPO activity and histological analysis of neutrophil inflammation in colon. As shown in Fig. 10, 5-ASB, 5-ASDB, Ols-DB and Ols-DBP significantly decreased the MPO activity (*p* < 0.01) in colonic tissues, as compared with the TNBS group, and they were significantly better than the positive control.

### 3.9 Effects of prodrugs on levels of MDA, SOD, GSH and GSH-Px

**Fig. 11:**
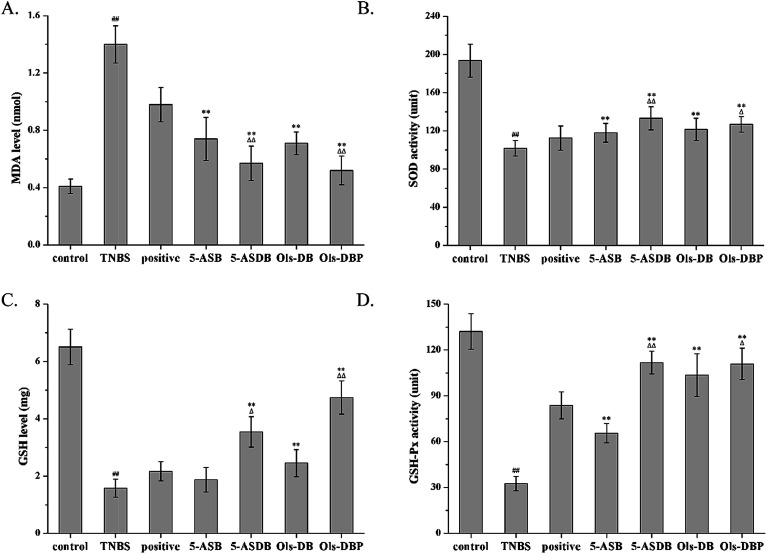
Effects of prodrugs on levels of MDA, SOD, GSH and GSH-Px. After treatment, the MDA levels (A), SOD activities (B), GSH levels (C) and GSH-Px activities (D) of mice in colonic tissues were measured to evaluate the potential therapeutic effects of prodrugs. SOD, superoxide dismutase; MDA, malondialdehyde; GSH, glutathione; GSH-Px, glutathione peroxidase. ^##^: *p* < 0.01 *vs.* control group; **: *p* < 0.01 *vs.* TNBS group; △: *p* < 0.05, △△: *p* < 0.01 *vs.* positive control group. Data are shown as mean ± SD, *n* = 12.

As compared with the control group, mice in the TNBS group showed a significantly higher MDA level (*p* < 0.01), lower GSH level and lower activities of SOD and GSH-Px (*p* < 0.01) (Fig. 11). After treated with different drugs, 5-ASB, 5-ASDB, Ols-DB and Ols-DBP significantly decreased the MDA levels in the colon of mice (*p* < 0.01) and increased the SOD and GSH-Px activities (*p* < 0.01), compared with the TNBS group. Mice in 5-ASDB, Ols-DB and Ols-DBP groups showed significantly higher levels of GSH than that of the TNBS group (*p* < 0.01). As compared with the positive control, 5-ASDB and Ols-DBP showed significantly greater effects on decreasing the MDA level (*p* < 0.01), and increasing the activities of SOD and GSH and the GSH-Px level in the colon of mice.

## 4 Discussion

5-ASA and butyrate are proved to have therapeutic effects on UC. However, 5-ASA is rapidly and nearly completely absorbed from the upper intestine when administrated orally. Butyrate undergoes rapid metabolism and is limited by its low oral bioavailability and short half-time (*t*_1/2_ = 6 min). Therefore, this study synthesized four mutual prodrugs through linking butyrate to 5-ASA or olsalazine. Utilizing the alkaline environment in colon and the hydrolysis of azoreductase, 5-ASA and butyrate are released from the prodrugs to achieve the colon-targeting effects. The synthesis processes of 5-ASB and Ols-DB were similar to that of aspirin, the modified processes are convenient to operate with easy post-treatment, less side-reactions and high yield. The synthetic routes of 5-ASDB and Ols-DBP were initially designed to use the nontoxic glycerin as a linkage, in order to reduce by-products, the 1,3-dihydroxyacetone was used in the final scheme. For the same reason, 5-nitrosalicylic acid was used instead of 5-ASA as the starting material. The yield of Ols-DBP was relatively low (49.5%), possibly because the high steric hindrance of olsalazine is likely to produce mono-substituted by-products and decrease the yield of the final product.

The lipophilicity and aqueous solubility of drugs are associated with their colon-targeting property, passive absorption from the gastrointestinal tract and their penetrability through the blood–brain barrier. In this study, the lipophilicity of drugs was evaluated by the widely used partition coefficients between octanol and water, the solubility of drugs was determined as equilibrium solubility. As compared with 5-ASA, the solubility of the four prodrugs were increased, while their partition coefficients were decreased, suggested that the absorption of the four prodrugs from the upper gastrointestinal tract and their systemic absorption would be reduced.^26^ Prodrugs would be directly delivered to the colon, thus achieving the colon-targeting property.

An important parameter determining the potential capacity of colon-targeting prodrugs are their hydrolysis behaviors under the environment of gastrointestinal tract. As the stability studies suggested that, 5-ASB was very stable in stomach contents and small intestine contents of rats, and hardly hydrolyzed (6.79%) after *in vivo* incubation in rat colons. It was possibly because the ester bond in 5-ASB was remarkable stable and hard to fracture due to the steric hindrance of its molecular structure. On the contrary, Ols-DB showed extreme instability in the *in vitro* and *in vivo* incubation studies, it would be rapidly and almost completely hydrolyzed in the gastrointestinal tract. The difference of stability between 5-ASB and Ols-DB might be related to the space structure of azo bond in Ols-DB. Therefore, the Ols-DB would be given to mice as an enema instead of by gavage in the following pharmacodynamics studies. 5-ASDB and Ols-DBP showed similar hydrolysis behaviors in the incubation studies. After incubated in stomach contents for 2 h, nearly half of them were hydrolyzed. Besides, they were hardly hydrolyzed in the small intestines but completely hydrolyzed in the rat colons. Therefore, the main influencing factor on the stability of 5-ASDB and Ols-DBP was the acid environment in stomach. In our further study, coating materials are taken into consideration to protect 5-ASDB and Ols-DBP from decomposing in the acid environment and enhance their colon-targeting properties.

The ameliorative effects of prodrugs on the TNBS-induced experimental colitis were evaluated by a series of indicators, which are effective in reflecting the inflammation in mouse models. The weight changes and DAI score of mice were used for real-time monitoring the state of mice in each group, and estimating the onset time of drugs. The survival rates, colon index and CDS could reflect directly the degrees of inflammation in mice after treatment. The activities of MPO, SOD and GSH-Px, and the levels of MDA and GSH in colonic tissues could reflect quantitatively the colitis of mice, while assisting in speculating the pathogenesis of the TNBS-induced colitis and the action mechanism of drugs.

During the experiment, the death of mice was found in all the test groups, especially in the 2^nd^ day after the induction of colitis. After dissected the dead mice, it could be observed that the major cause of death in mice was ileus caused by severe colitis, some of the dead mice showed enterobrosis, some even leaded to severe adhesion of the intestinal mucosa and it was unable to separate the colons. The TNBS-induced mouse models were found to have bloody stools, diarrhea, reducing diet, hypokinesia, and with different degrees of huddle and weight loss. After treated with different drugs, symptoms of the surviving mice in test groups were gradually ameliorative, all the indicators were began to close to the control group.

The DAI of mice in test groups were continuously increased in the 2 days after the induction of colitis, and started falling at different degrees from the 3^rd^ day. After 5 days of treatment, mice administrated with the four prodrugs showed significantly decrease of the DAI, which were even closed to that of the control group. Ols-DB showed the most significant effects in the first 4 days and became equivalent to the rest of the three prodrugs at the end of the treatment, probably because Ols-DB was given as an enema rather than gavage administration. MPO is a major constituent of neutrophil cytoplasmic granules, the total activity of MPO is a direct measure of neutrophil sequestration in tissues. As been reported by Krawisz *et al.*, the determination of MPO activity in the intestine is a simple biochemical assay that can be used to quantitate inflammation, thus MPO is a useful marker of acute inflammation in colonic tissues from animals with experimental colitis.^27^ The increased level of MPO in colon homogenate reflects the neutrophilic leukocytosis, the presence of inflammation, and the degrees of neutrophil infiltration. The reduction of MPO activity can be interpreted as a manifestation of the anti-inflammatory property of the given drugs. In this study, the four prodrugs could significantly decrease the MPO activities as compared with the TNBS group, besides, they had greater effects than the positive control.

SOD is a kind of enzyme that alternately catalyzes the dismutation of the toxic superoxide radical into either ordinary molecular oxygen or hydrogen peroxide. Superoxide is produced as a by-product of oxygen metabolism and causes many types of cell damage.^28^ Thus, SOD is an important antioxidant defense in nearly all living cells exposed to oxygen. The SOD activities in colons reflect directly the degree of damages and active inflammation, and the development of inflammation over a period of time. MDA is a reactive aldehyde formed naturally by lipid peroxidation of polyunsaturated fatty acids, and it is one of the reactive electrophile species that cause toxic stress in cells and form covalent protein adducts. The production of MDA is used as a biomarker to measure the level of oxidative stress in an organism.^29^ Therefore, the degree of lipid peroxidation can be estimated by the levels of MDA in tissues, and reflect indirectly the degree of cellular damage. GSH is one of the major endogenous antioxidants produced by cells with multiple functions, including preventing damage to important cellular components caused by reactive oxygen species, participating directly in the neutralization of free radicals and reactive oxygen compounds, and maintaining exogenous antioxidants in their reduced forms.^30^ The level of GSH in a tissue is used as an indicator to evaluate its antioxidant ability, therefore, reflecting directly the degree of inflammatory damage in the colons. GSH-Px is an enzyme family with peroxidase activity whose main biological role is to protect the organism from oxidative damage.^31^ The biochemical function of GSH-Px is to reduce lipid hydroperoxides to their corresponding alcohols and to reduce free hydrogen peroxide to water, thus maintaining the integrity and function of cell membrane.

In this study, the therapeutic effects of prodrugs on the TNBS-induced experimental colitis were evaluated through their ability to increase the activities of SOD, GSH and GSH-Px, as well as decrease the MDA levels in colonic tissues. Data of the pharmacodynamics studies showed that the four synthesized prodrugs (5-ASB, 5-ASDB, Ols-DB and Ols-DBP) all had certain ameliorative effects on the TNBS-induced colitis in mice as compared with the control group, 5-ASDB and Ols-DBP were significantly greater than the positive control, Ols-DB was used as an enema and could be just as effective as 5-ASDB and Ols-DBP. Besides, the results suggested that the anti-colitis efficacy of the synthesized prodrugs were strongly associated with their ability of anti-oxidative damage, but further study is required to confirm the mechanism in detail.^32,33^ Currently, studies are made to improve the colon-targeting property of the prodrugs, such as using pH-sensitive materials to prepare coating tablets or capsules.

## 5 Conclusions

Four novel mutual prodrugs of 5-ASA and butyrate were designed and synthesized in this study, their chemical structures were confirmed by DSC, ^1^H NMR, FTIR and MS. The lipophilicity of the prodrugs was determined in the *n*-octanol/phosphate buffer system and the aqueous solubility was referred as their equilibrium solubility, as compared to 5-ASA, the synthesized prodrugs had higher solubility and lower partition coefficients. The hydrolysis behaviors of the prodrugs were evaluated by the stability studies *in vitro* and *in vivo*, as a result, 5-ASB was very stable but Ols-DB showed extreme instability in the environment of gastrointestinal tract, 5-ASDB and Ols-DBP showed desirable colon-targeting property. The ameliorative effects of the prodrugs on TNBS-induced colitis in mice were evaluated by a series of indicators. Data showed that all of the four prodrugs had certain therapeutic effects. 5-ASDB and Ols-DBP were significantly greater than the positive control when orally administrated to mice. Ols-DB was used as an enema and could be as effective as 5-ASDB and Ols-DBP. Besides, results suggested that the anti-colitis efficacy of the synthesized prodrugs might be associated with their ability of anti-oxidative damage.

## Conflicts of interest

There are no conflicts to declare.

## Supplementary Material
